# Urgent endovascular ligature of a ruptured splenic artery pseudoaneurysm in a patient with acute pancreatitis: a case report

**DOI:** 10.1186/1752-1947-9-6

**Published:** 2015-01-09

**Authors:** Anna Maria Ierardi, Mario Petrillo, Raffaella Capasso, Federico Fontana, Alessandro Bacuzzi, Ejona Duka, Domenico Laganà, Gianpaolo Carrafiello

**Affiliations:** 6 Department of Radiology, University of Insubria, Ospedale di Circolo e Fondazione Macchi, Viale Borri 57, 21100 Varese, VA Italy; 7 Department of Radiology, Second University of Naples, P.za Miraglia, 2, 80138 Naples, NA Italy; 8 Anaesthesia and Palliative Care, University of Insubria, Ospedale di Circolo e Fondazione Macchi, Viale Borri 57, 21100 Varese, VA Italy

**Keywords:** Acute pancreatitis, Contrast-enhanced ultrasonography, Endovascular ligature, Impaired renal function, Pancreatic pseudoaneurysms

## Abstract

**Introduction:**

We report on the successful endovascular treatment of a ruptured splenic artery pseudoaneurysm. Our patient had acute pancreatitis superimposed on chronic calcific pancreatitis and chronic renal impairment. Contrast-enhanced ultrasonography was used to assess post-embolization results.

**Case presentation:**

Our patient was a 67-year-old white Caucasian man with recurrent pancreatitis. Computed tomography angiography showed a pancreatic pseudocyst with a ruptured pseudoaneurysm, which was successfully embolized using an endovascular percutaneous approach. At six months, persistent renal failure led to contrast-enhanced ultrasonography. This confirmed the absence of turbulent blood flow and extravasation of contrast medium in the pseudocyst.

**Conclusion:**

Our experience with this case leads us to support the role of interventional radiology as a first-line treatment tool. Contrast-enhanced ultrasonography can be used to follow-up embolization procedures in patients with impaired renal function.

## Introduction

Severe pancreatic inflammation and necrosis, which cause the local spread of pancreatic fluids rich in exocrine enzymes, may cause elastolytic erosions of the vessel wall, representing one of the causes of vessel injury [1–4]. This can lead to vascular complications, such as the formation of a pseudoaneurysm if there is bleeding into a contained space or organ, a contained hematoma if the pseudoaneurysm becomes thrombosed or active extravasation stops, or frank intraperitoneal hemorrhage if the pseudoaneurysm ruptures. Pancreatic pseudoaneurysm (PSA) is a rare vascular complication of pancreatitis, with an incidence up to 10% in patients with chronic disease. It commonly results from the erosion of the pancreatic or peripancreatic arteries into a pseudocyst, creating a permanent communication between the vasculature and the pseudocyst itself [1].

In the setting of chronic pancreatitis causing pseudoaneurysm, the most commonly involved artery is the splenic artery (60% to 65%), followed by gastroduodenal (20% to 25%), pancreaticoduodenal (10% to 15%), hepatic (5% to 10%) and left gastric arteries (2% to 5%). Less frequently involved are the dorsal pancreatic, hepatic and superior mesenteric arteries [2]. These vascular complications may have an unpredictable evolution and can have lethal consequences caused by rupture and massive bleeding. Mortality can be 12.5% in treated patients and higher than 90% in untreated patients [3, 5]. Historically, pseudoaneurysms secondary to pancreatitis were treated surgically, with an associated mortality of approximately 56%. Thanks to the advances in interventional radiological techniques, the paradigm has largely shifted towards endovascular treatment.

We report the successful endovascular treatment of a PSA in a patient with acute pancreatitis superimposed on chronic calcific pancreatitis and renal impairment. Our experience with this case leads us to support the first-line role of urgent endovascular ligature confirmed by the use of contrast-enhanced ultrasonography (CEUS) in patients with impaired renal function [6]. The report has been approved by our Internal Review Board and our patient has given his consent.

## Case presentation

A 67-year-old white Caucasian man with chronic pancreatitis, cholelithiasis and impaired renal function presented to our emergency department for anorexia and moderate to intense pain in his epigastrium radiating to his back. His usual analgesics had not lessened the pain, which had worsened in the previous four days. On physical examination he was cachectic, pale, oriented and apyretic (36.8°C). He had normal heart sounds (78 beats per minute) and a slightly high arterial pressure (147/80mmHg). His abdomen was particularly tender in the left upper epigastric area and the tenderness increased with palpation. Laboratory tests revealed the following data: leukocytosis at 11×10^−3^/μL, low hemoglobin level of 8.1g/dL, platelet count of 258×10^−3^/μL, serum creatinine of 2.35mg/dL, lipase level of 420IU/L, amylase of 220IU/L, lactate dehydrogenase of 450IU/L, glucose of 76mg/dL, aspartate aminotransferase of 45IU/L and total bilirubin of 1.1mg/dL. He had no history of previous upper gastrointestinal bleeding.

**Figure 1:**
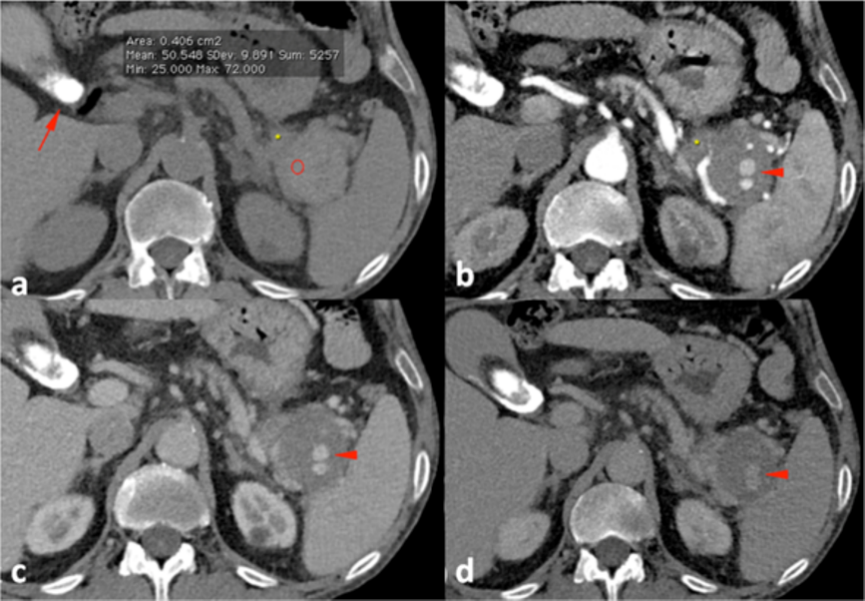
**Initial radiology findings. (a)** Non-enhanced computed tomography showing a thin, dense imbibition of fat around the pancreatic tail (asterisk) near a 5.1cm pseudocyst with hyper-dense content and a gallstone inside the gallbladder (arrow). **(b-d)** Contrast-enhanced computed tomography shows a pseudoaneurysm arising from a splenic artery branch inside the pseudocyst.

We performed contrast-enhanced computed tomography (CECT), which showed a normally enhancing pancreas, with enhancement comparable to that of his spleen even though the head and body of the pancreas were slightly enlarged with subtle peripheral fat-stranding. Prior to contrast medium (CM) administration, subtle calcifications had been visible throughout the pancreas body. These findings were suggestive of edematous, interstitial pancreatitis superimposed on chronic calcific pancreatitis. A 5.1cm pseudocyst was visible in the pancreatic tail, characterized by hyper-dense content in the non-enhanced contrast scan (mean density 54 Hounsfield units, standard deviation 13.8), that did not enhance after CM administration. We first suspected proteinaceous content, but then detected enlarged, twisty, dilated vessels within the cyst, arising from a branch of the patient’s splenic artery. This was highly suspicious for the presence of a bleeding pancreatic PSA. On the basis of the density values of the pseudocyst without and after intravenous CM administration (Figure 1a-d), we could not exclude a recent bleed.

**Figure 2:**
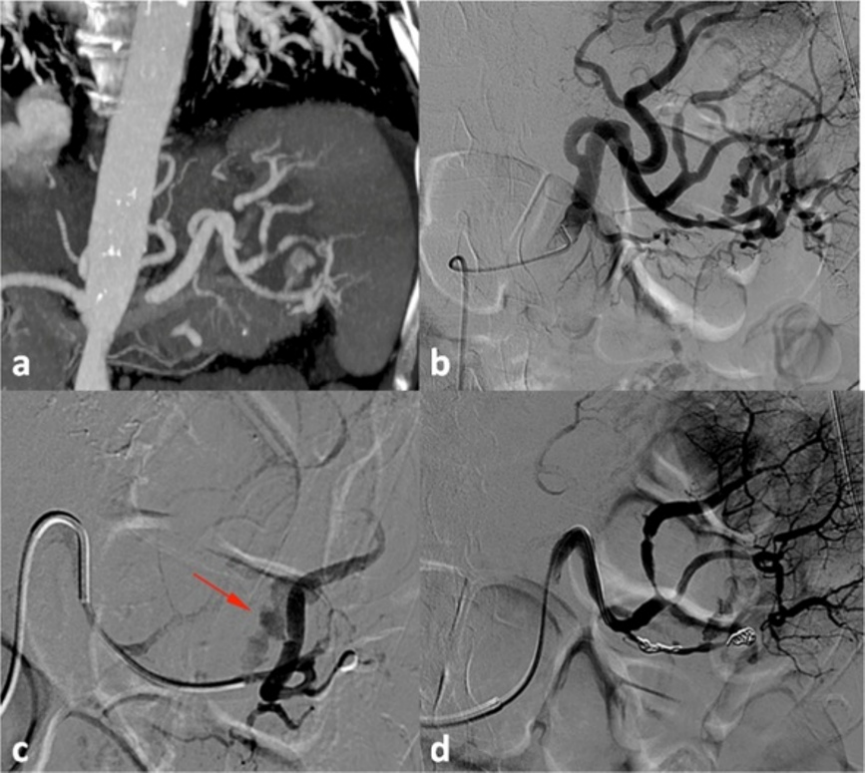
**Angiogram images. (a)** Multiplanar reformation reconstructions demonstrating a pseudoaneurysm arising from an inferior polar splenic artery branch. **(b,c)** Selective and super-selective angiogram confirming the pseudoaneurysm and its bleeding (arrow). **(d)** Final angiogram showed the presence of distal and proximal metallic microcoils after their deployment.

The possibility of surgical hemostasis was discarded by surgeons because of his multiple severe systemic illnesses and very poor general performance. He had severe pulmonary hypertension due to an advanced idiopathic interstitial fibrosis, and severe impaired renal function (estimated glomerular filtration rate (eGFR) using the Modification of Diet in Renal Disease (MDRD)^a^ = 28mL/min/1.73m^2^. Because of his low hemoglobin level, an emergency celiac angiography was planned after collegial discussion with surgeons to exclude an active bleeding arising from the PSA. A splenic angiogram showed an active extravasation of CM originating from the PSA, which showed well after a super-selective catheterization obtained with a 2.7-F coaxial micro-catheter (Progreat, Terumo, Tokyo, Japan). Therefore, an endovascular ligature was tried as first-line therapy. Considering the tortuosity of his splenic artery, the micro-catheter was placed into his splenic artery distal to the pseudoaneurysm to achieve distal embolization with three (two 5mm and one 6mm) metallic microcoils (VortX; Boston Scientific, Natick, MA, USA). After micro-catheter retraction proximal to the pseudoaneurysm, two (one 5mm and one 6mm) metallic microcoils (VortX) were deployed (Figure 2a-d). Angiography after endovascular ligature showed exclusion of the PSA with stagnation of CM within the pseudocyst (Figure 2a-d).

**Figure 3:**
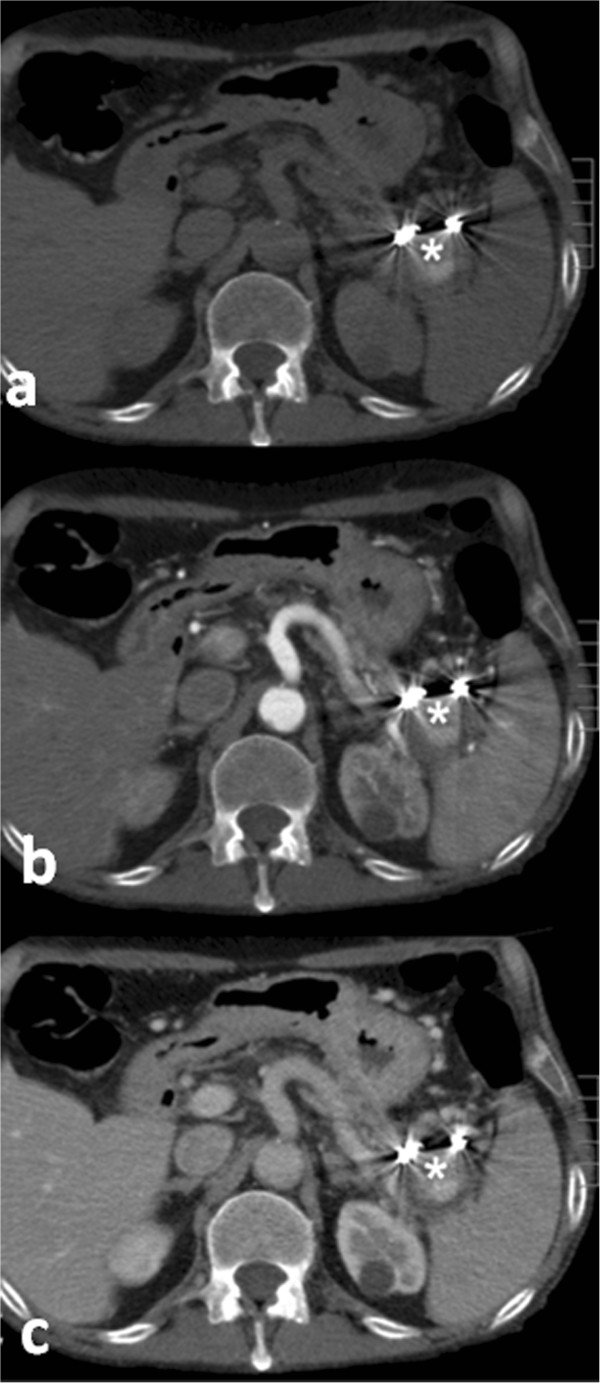
**Post-endovascular ligature computed tomography images.** Computed tomography performed **(a)** without intravenous contrast medium administration and **(b,c)** after administration shows microcoils deployed in the splenic artery, with stagnation of contrast medium inside the pseudocyst (asterisks in **a**) and exclusion of the pseudoaneurysm in the arterial **(b)** and venous **(c)** phase.

Our patient’s clinical course was acceptable with progressive hemoglobin recovery up to 11.4g/dL 48h after the transarterial embolization. Wide-spectrum antibiotics and analgesic and antipyretic drugs were administrated to prevent infectious complications and to control pain. Six days after the procedure, CECT confirmed the complete exclusion of the splenic pseudoaneurysm with the same ‘stagnation’ of CM (Figure 3a-c) that was appreciable on the post-embolization angiogram. Some ischemic damage of the spleen was identified, but most of the splenic parenchymal vascularization was present.

**Figure 4:**
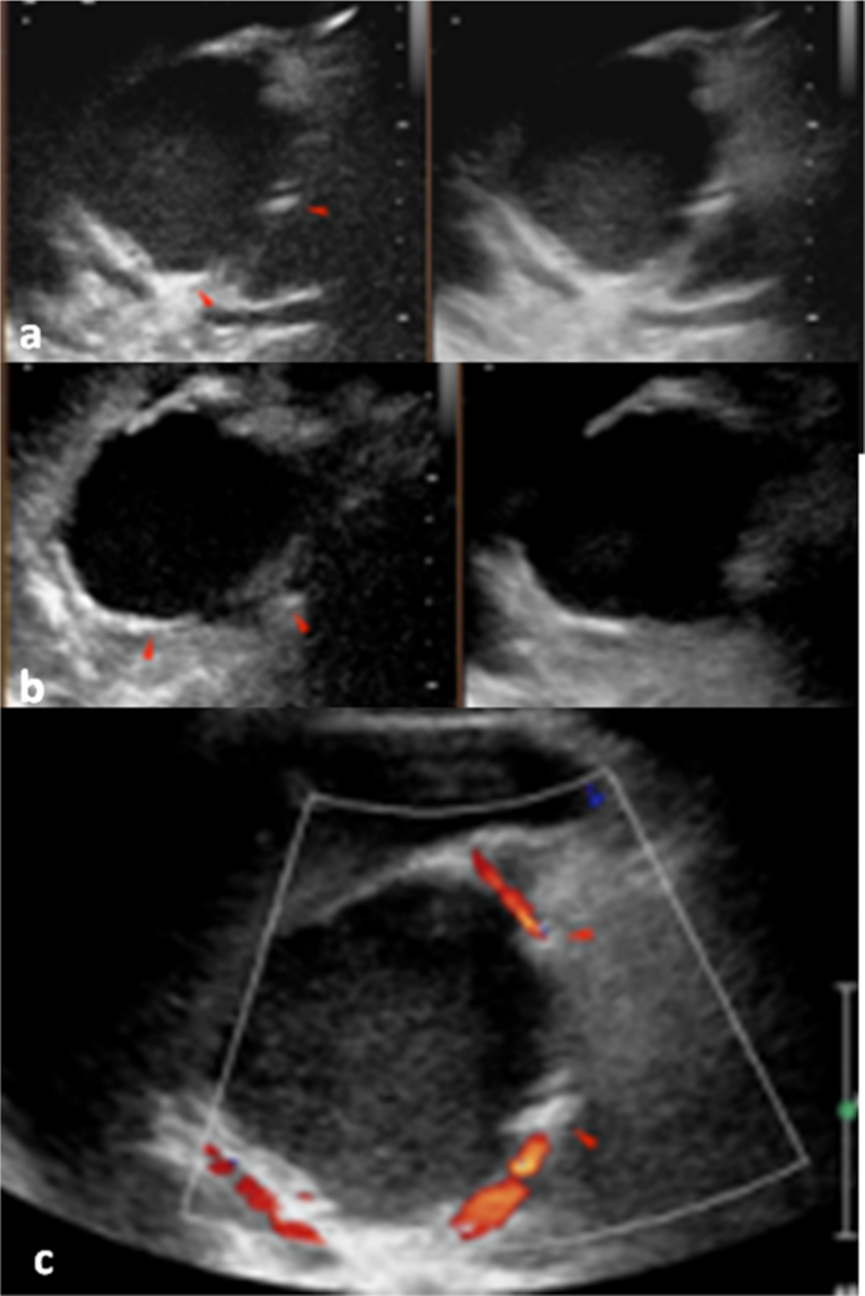
**Contrast-enhanced ultrasonography performed after six months. (a-c)** Shows the presence of deployed metallic microcoils (arrowheads, **a-c**) and the absence of reperfusion of the pseudocyst after intravenous contrast medium administration.

Our patient was discharged in a stable condition. Follow-up at three and six months comprised combined color Doppler ultrasound with CEUS using a dedicated CM (Sonovue, Bracco, Italy) (Figure 4a-c) because of the persistent elevation of his serum creatinine levels (1.7mg/dL and eGFR with MDRD = 40mL/min/1.73m^2^). This choice was concordant with European Society of Urogenital Radiology (ESUR) guidelines [6] to consider an imaging method that does not use iodine-based CM in order to reduce the risk of a CM-induced nephropathy (CIN) in a patient at high risk (eGFR lower than 45mL/min/1.73m^2^). Both examinations confirmed the presence of a pancreatic pseudocyst with a slightly increased diameter (60mm) without signs of active bleeding inside the cyst (Figure 4a-c).

## Discussion

Hemorrhage is a rare but potentially fatal complication in pancreatitis, with a greater incidence observed in chronic disease (7% to 10%) than in acute (1% to 6%) [2]. Pseudoaneurysms may cause bleeding if they rupture into the peritoneal cavity, retroperitoneum or adjacent structures, such as bowel (presenting as massive gastrointestinal hemorrhage) or pancreatic duct of Wirsung (presenting as hemosuccus pancreaticus) [4]. In the detection of vascular injuries due to pancreatitis, CECT and, less frequently, ultrasonography (US) represent non-invasive and suitable first-line imaging modalities. In our case, CECT detected the presence of a PSA, while angiography revealed its bleeding and permitted its embolization. One significant advantage of angiography is in the evaluation of the arterial bed in real-time, allowing an assessment of the vascular supply and collateral circulation. This information is required to plan treatment. Moreover, angiography may identify other smaller pseudoaneurysms not seen by US or CECT. It can also help discriminate PSA from other lesions, such as true aneurysms, arteriovenous fistulas and vascular malformations, which can have similar appearances on other imaging modalities. Because of its high sensitivity (100%) in the detection of arterial bleeding, angiography remains the diagnostic gold standard when endovascular treatment is indicated [2–6].

The natural course of PSA is unpredictable, and so treatment is recommended [5]. In the management of PSAs several factors have to be considered, including hemodynamic stability, coagulation status and source of bleeding. Traditionally, surgery was the treatment of choice, including arterial ligation, direct intra-pseudocystic ligation, and resection of a part of the pancreas. The morbidity of surgical interventions has been reported between 16% and 50% [2]. With the advancement of endovascular techniques, endovascular ligature has become the first-line treatment [2]. In some cases, transarterial catheter embolization can be performed before surgery to stop bleeding, as surgery sometimes is not indicated in emergent conditions in patients with hemodynamic instability and hemorrhagic shock [5]. However, endovascular therapy remains the first-line option for known arterial bleeding (preferably in a patient with a stable hemodynamic status) and is considered a safe and effective modality to treat visceral pseudoaneurysms [4, 7]. Metallic coil embolization is preferable distal and proximal to the site of arterial extravasation (the so-called sandwich technique, or endovascular ligature), thereby preventing backflow from the collateral circulation [4, 7, 8]. According to data in the literature, endovascular ligature was the fastest way to arrest bleeding in our patient and may be considered a life-saving procedure. We controlled ischemic damage of the spleen allowing the revascularization of the splenic parenchyma by collateral branches. Any infectious complications were prevented with antibiotic prophylaxis.

An important aspect emerging in the follow-up of a ligated PSA in a patient with impaired renal function is the choice of CEUS. According to ESUR guidelines [6], in the planning of elective examinations, it is mandatory to select an imaging method that does not use iodine-based contrast in a patient at high risk for a CIN. Adverse events (such as nephropathy) for sonographic contrast agents have not been reported in the literature [6, 9–11]. Our experience with this case underlines the rare possibility of a vascular complication in a patient with chronic pancreatitis. When clinical data and laboratory tests are suggestive for a vascular complication, an urgent diagnosis is necessary. An early diagnosis may be life-saving for the patient. An endovascular approach is now usually the first choice of treatment; another aspect to bear in mind is the importance of an endovascular ligature of the PSA to account for the presence of multiple collateral branches that may refill the PSA. Moreover, the role of CEUS during follow-up in asymptomatic and stable patients should be emphasized.

## Conclusion

Our case represents an example of successful management of a rare vascular complication in a patient with chronic pancreatitis and several important comorbidities. Our experience supports the first-line role of interventional radiology, and demonstrated a successful outcome of an endovascular ligature without the need for surgery. We encourage the use of CEUS to follow-up embolization procedures in patients with impaired renal function.

## Consent

Written informed consent was obtained from the patient for publication of this case report and any accompanying images. A copy of the written consent is available for review by the Editor-in-Chief of this journal.

## Endnote

^a^For the MDRD formula, see http://mdrd.com/.

## Abbreviations

CEUS: contrast-enhanced sonography
CECT: contrast-enhanced computed tomography
CIN: contrast medium-induced nephropathy
CM: contrast medium
eGFR: estimated glomerular filtration rate
ESUR: European Society of Urogenital Radiology
MDRD: Modification of Diet in Renal Disease
PSA: pseudoaneurysm.
